# Characterization of C-reactive protein in dogs undergoing medial patellar luxation surgery

**DOI:** 10.1371/journal.pone.0231445

**Published:** 2020-05-08

**Authors:** Marte Jervan, Donald A. Szlosek, Hanne Friis, Michael J. Coyne, Dennis DeNicola, Ole H. Johnsen

**Affiliations:** 1 Fredrikstad Dyrehospital, Fredrikstad, Norway; 2 IDEXX Laboratories, Inc., Westbrook, Maine, United States of America; East Carolina University Brody School of Medicine, UNITED STATES

## Abstract

C-reactive protein (CRP) is a major acute phase protein used to monitor response to treatment during surgical recovery. Depending on the anatomical problem, surgery type and technique, the level of CRP can change drastically. The aim of this study was to describe the changes in CRP and white blood cell (WBC) levels following surgery for medial patellar luxation in otherwise healthy dogs. Twenty-two dogs completed the study. CRP was measured using a commercially available dry chemistry slide on a commercially available in-clinic analyser. Analyses were performed using the Wilcoxon Rank Sum test and a mixed effects Poisson regression model. A significant change in CRP levels was found between the pre-anesthetic and 24 hr post-surgical timepoint with a median difference of 92.0 mg/dL (P < 0.001). Though a median drop in the CRP value of 13.9 mg/dL was observed between the 24 hr and 48 hr post-surgical time period, the result was not statistically significant (P = 0.456). Similarly, there was a significant increase in WBC between the pre-anesthetic and 24-hr post-surgical time point (P < 0.001) followed by a significant decrease in WBC between the 24 hr and 48-hr post-surgical time points (P = 0.015). In this study population, CRP levels were observed to aid in monitoring of the overall health of the dogs following surgery for medial patellar luxation. The results of this study suggest that both CRP and WBC values significantly increase by 24 hr but where CRP levels remain elevated through 48 hr post-surgery, WBC showed a significant drop between 24 and 48 hr. Further investigation into the length of time for both CRP and WBC to reach basal levels in this particular type of surgery would be of value to monitor recovery from surgery.

## Introduction

C-reactive protein (CRP) is a major acute phase protein (APP) that can be used to monitor response to treatment during surgical recovery as a supplement to bone marrow response. CRP increases following an inflammatory stimulus (e.g. surgery) and has been observed to peak within 24 to 48 hr post injury [1,2]. A rapid decrease in CRP is seen following resolution of the inflammatory process, making it a valuable indicator for monitoring post-surgical recovery. Depending on the anatomical location, surgical type and technique used during surgery, CRP concentrations can change markedly [3,4]. In a number of orthopaedic surgical studies, changes in CRP levels as high as 800 percent have been observed [3]. The primary aim of this study was to describe the changes in CRP levels following elective surgery for medial patella luxation with trochlear block recession in combination with tibial tuberosity transposition in otherwise healthy dogs from a baseline value obtained pre-anesthesia through the first 48 hr post-surgery.

## Materials and methods

Twenty-nine dogs, presented to Fredrikstad Dyrehospital, Norway (FD) between April, 2017 and August, 2018 for surgical correction of medial patellar luxation were initially recruited into the study. Each surgery was given a unique surgical identification (ID) number and all surgeries were performed by one of two experienced surgeons at the hospital using exactly the same surgical procedure. For dogs who underwent two separate surgeries in the study, a second surgical identification number was given for each surgery. Surgical methods used in this study were trochlear block recession in combination with capsulorrhaphy and tibial tuberosity transposition [5,6]. A full description of the surgical and anesthetic protocols can be found in S1 Appendix. The dogs underwent a complete clinical examination and were determined to be healthy other than having a patellar luxation. Body weight and body condition scores were recorded and only patients evaluated clinically as healthy progressed to having pre-anesthetic blood work performed. Blood for pre-anesthetic screening was collected from the cephalic or jugular vein depending on the size of the patient and then transferred to EDTA anticoagulant tubes (1 mL) for a complete blood count (CBC) and plain serum tubes (2 mL) for chemistries and canine CRP measurements. Sampling technique, handling and running the samples followed the standard operating procedures at the hospital. Blood sample collection was scheduled during routine follow up of the hospitalized dog at 24 (± 2 hr) and 48 (±2 hr) post-surgery. No blood samples were taken beyond 48 hr post-surgery. CRP was measured in FD using a commercially available dry chemistry slide (IDEXX Catalyst^®^ CRP Test) with a commercially available in-clinic analyser (IDEXX Catalyst One^®^)[7]. Routine clinical chemistry and haematology profiles were processed in FD using in-clinic analysers (the Catalyst One^®^ and IDEXX ProCyte Dx^®^ Haematology Analyzer) within 4 hr of sample collection. All blood samples were obtained on written consent of the pet owner. Reference intervals for WBC (5.05–16.76x10^3/L) and CRP (0.1–10 mg/L) in canines were previously established according to Clinical Laboratory and Standards Institute (CLSI) guidelines [8].

### Statistical analysis

A Wilcoxon signed ranked sum test was used to compare the difference in CRP concentrations across all time points. Differences in white blood cell (WBC) counts were tested as a generalized linear mixed model using the Poisson distribution and surgical ID as a random effect. The Cameron & Trivedi test was used to test for over dispersion [9]. Sex-related effects were also analysed for WBC count and CRP with the regression methods mentioned above. Due to the small number of neutered males (n = 2) in the study, both neutered (NM) and intact males (IM) were collapsed into a single male variable. No neutered females (FN) were enrolled in the study, thus all comparisons were made to intact females (FI). Inflammation markers (CRP and WBC) were reported as median and interquartile range for all time points. Graphical analysis was reported as both boxplot and line graph by subject ID for all three parameters. All data analysis was performed with R version 3.3.1 [10]. Regression analysis was performed using the *lme4* package [11]. Graphical analysis and data cleaning was performed using the *dplyr*, *ggsignif*, and *ggplot2* R packages [12–14].

## Results

Of the 29 dogs with medial patellar luxation initially enrolled in the study, five were excluded from the study because of insufficient blood-sampling. One dog was excluded because of severe haemorrhagic gastroenteritis that developed within the first 24 hr after surgery. One dog was excluded because of an elevated serum C-reactive protein level of 50 mml/L detected in the pre-anesthetic blood sample. No final diagnosis of the cause of inflammation was made. A total of 23 surgeries were performed with one subject having two separate surgeries performed 6 months apart (surgical ID # 9811 and #9836). The first of the two surgeries (surgical ID #9811) did not have adequate blood sample or haematology analysis and thus was excluded from study analysis. Breeds included Bichon frise (n = 3, 14.3%), Kleinspitz (n = 3, 14.3%), Chihuahua (n = 2, 9.5%), Jack Russell Terrier (n = 2, 9.5%), and one of each of the following breeds: Boston terrier, English toy terrier, Japanese spitz, Labrador retriever, and Petit Brabancon. Six subjects were of mixed breed. There were 15 (65.2%) intact females, 6 intact males (26.1%), and 2 (8.7%) neutered males. The median weight was 5.5 kg (range: 1.6–30.6 kg). The median body condition score was 5 (range: 4–7) out of a 9-point scale. Out of 22 surgeries, nine had missing data for duration of surgery. The median duration of surgery was 37.0 minutes (range: 24–45 minutes). WBC count was missing for a single subject on the 48-hr post-surgery time point (surgical ID #9813).

**Fig 1:**
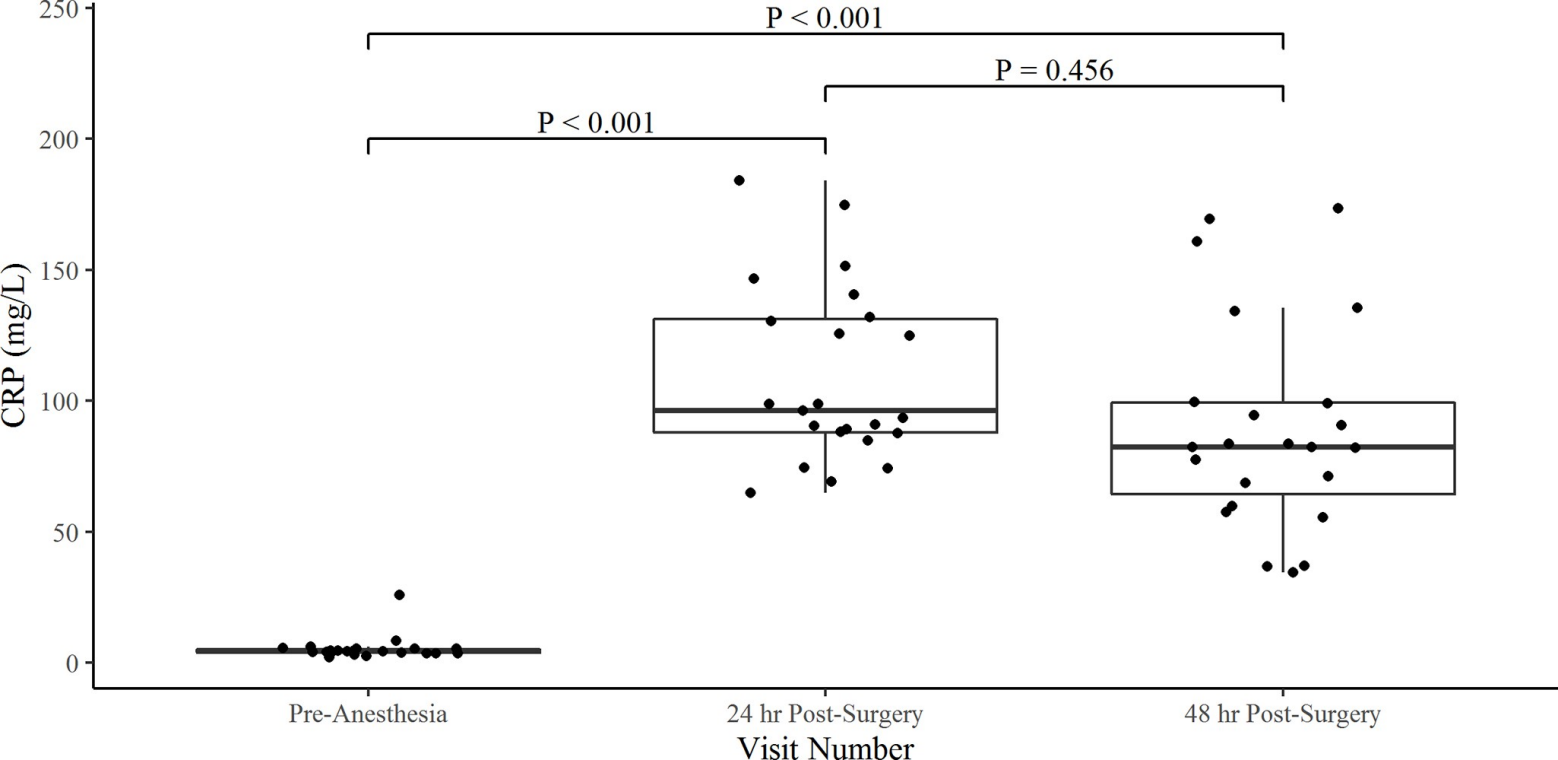
Box and whisker plots for the comparison of CRP levels pre- and post- medial patellar luxation surgery. The box represents the first quartile (Q1), median, and third quartile (Q3). The whiskers represent the upper and lower bound of 1.5x the inter-quartile range (IQR).

A significant change in CRP levels was found between the pre-anesthetic and 24 hr post-surgical timepoint with a median difference of 92.0 mg/dL (P < 0.001, Fig 1, Table 1). Seven (30.4%) out of the 22 surgeries had an increase in CRP value between the 24- and 48-hr time point, but no statistical significance was observed (P = 0.456, Fig 1, S1 Fig). Though a median drop in the CRP value of 13.9 mg/dL was observed between the 24 hr and 48 hr time period, the result was not statistically significant (P = 0.456, Fig 1, S1 Fig). No association was found between gender and CRP during the pre-anesthetic time point (P = 0.619, ref = FI, the 24-hr post-treatment time point (P = 0.396, ref = FI), and the 48-hr post-surgery time point (P = 0.733, ref = FI).

**Table 1:** Biomarker values for each surgical time point.

	Pre-Anesthetic	24 hr Post-Surgery	48 hr Post-Surgery
	Median (IQR)	Median (IQR)	Median (IQR)
CRP (mg/L)	4.4 (3.8–5.2)	96.4 (87.8–129.3)	82.5 (62.1–99.5)
WBC (Count x 10^3^/μL)	8.1 (6.1–10.0)	14.3 (12.9–16.7)	11.3 (9.8–13.7)

**Fig 2:**
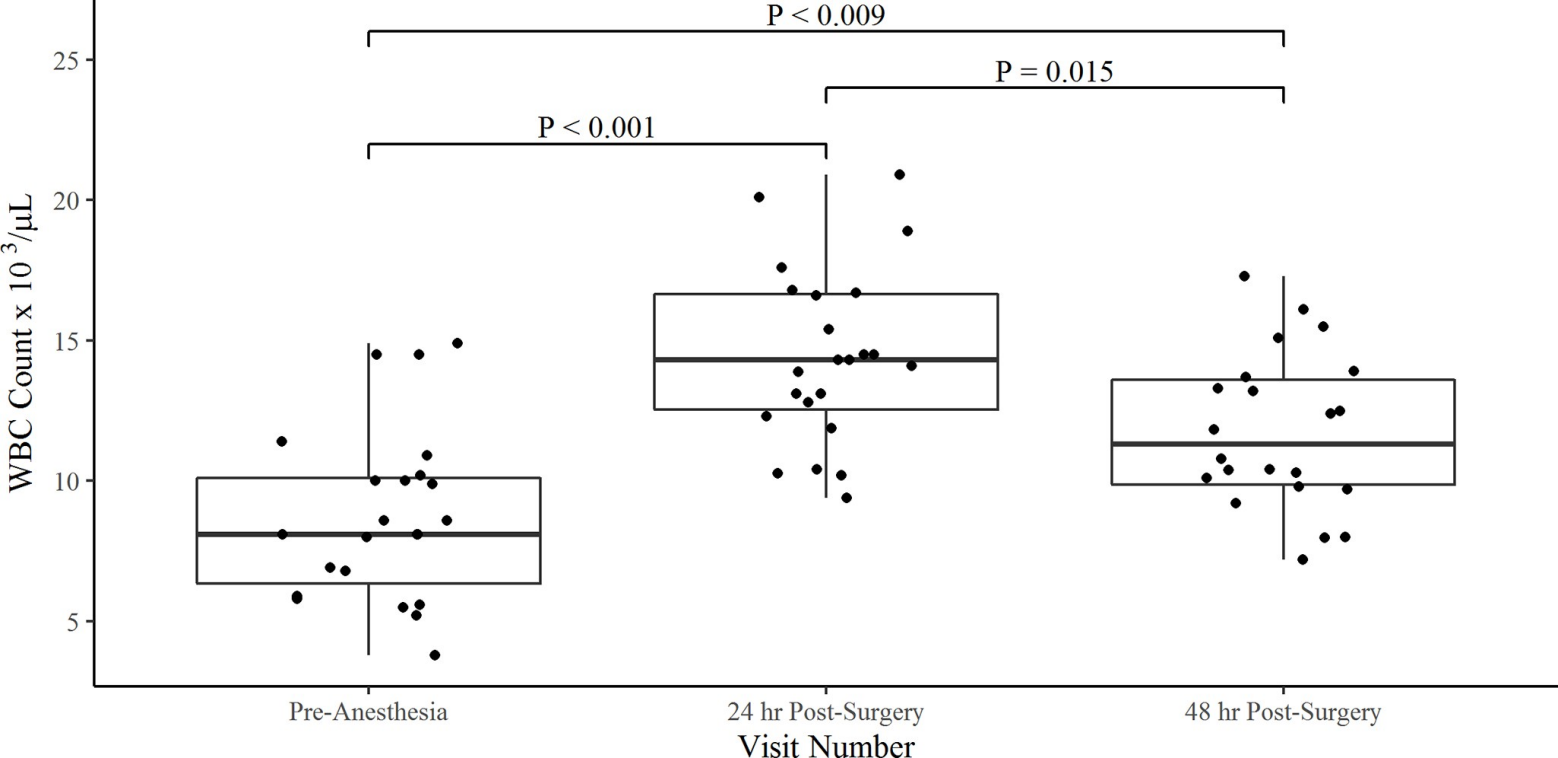
Box and whisker plots for the comparison of WBC counts pre- and post-medial patellar luxation surgery. The box represents the first quartile (Q1), median, and third quartile (Q3). The whiskers represent the upper and lower bound of 1.5x the inter-quartile range (IQR).

There was a significant difference in WBC between the pre-anesthetic and 24-hr post-surgical time point with a median increase of 6.2 x 10^3^ respectively (P < 0.001, Fig 2, Table 1). Between the 24 hr and 48-hr post-surgical time points a significant difference in WBC count was observed with a median drop of 3.0 x10^3^ (P = 0.015, Fig 2, Table 1). In addition, three dogs were observed to have an increase in WBC count between the 24 hr and 48-hr post-surgical time points (S2 Fig, S2 Dataset). No association was found between gender and WBC count during the pre-anesthetic time point (P = 0.294, ref = FI, the 24-hr post-treatment time point (P = 0.987, ref = FI), and the 48-hr post-surgery time point (P = 0.450, ref = FI).

## Discussion

The results of this study show that CRP levels peak at 24 hr post- medial patellar luxation surgery with a median value of 96.4 mg/L. CRP levels increased significantly with a median increase of 2,191% between baseline and 24 hr post-surgery. The CRP levels stayed persistently increased to 48 hr post-surgery with 30.4% of dogs undergoing surgery seeing an increase in CRP levels between 24 and 48 hr post-surgery. These results are in support of previous studies finding CRP levels peaking within 24–48 hr post-surgical intervention [2–4,15]. WBC were also observed to increase between the pre-anesthetic and 24 hr post-surgical time point. By the 48 hr time point, WBC counts were observed to drop significantly (P = 0.015) from the 24 hr time but remained higher than the pre-anesthetic time point.

With previous studies showing large variation in CRP depending on surgical technique and degree of tissue trauma [1], the standardization of the procedure at this hospital and the consistency in peri- and post- operative analgesics were all used to minimize the potential bias of different surgeons and different levels of expertise which could potentially influence the degree of inflammatory response.

In several dogs in this study, both CRP levels and WBC increased between the 24- and 48-hr time points. A previous study has shown that CRP levels will drop to baseline pre-anesthetic levels within seven days after laparoscopic colopexy[16]. In our study, seven of the dogs were observed to have an increase in CRP levels between the 24 and 48 hr time periods suggesting the acute-phase response may continue for longer after an otherwise uncomplicated surgical procedure. As no additional measurements were collected after 48 hr post-surgery, we were limited in the characterization of this response over a longer time. Another limitation of the study included the relatively low number of dogs as well as missing data points for some dogs, including haematology values and duration of surgery. Since eight patients did not have duration of surgery collected, we are limited in the assessment of the surgery duration.

## Conclusion

In this study population, CRP and WBC levels were observed to aid in monitoring of the overall health of the dogs after trochlear block recession in combination with tibial tuberosity transposition for dogs with medial patellar luxation. Obtaining baseline values to assess health status prior to invasive surgery may be of clinical value as any existing systemic inflammation in a patient undergoing elective orthopaedic surgery may be the cause of postponing the surgery to minimize the risk of post-surgical infections and other complications. The results of this study suggest that CRP values dramatically increase by 24 hr and remained elevated out to 48 hr post-surgery. WBC counts observed a similar increase 24 hr post-surgery, but were trending downward by 48 hr. Further investigation into the length of time for CRP and WBC to reach basal levels will aid following the recovery of the dogs. It should be noted that all WBC counts remained within the analyser reported reference intervals (5.05–16.76 x 10^3^/μL) throughout the duration of the study. Without comparison to baseline values, significant changes in WBC counts to help identify active inflammation would not be possible. In contrast, all CRP values were above the low end of the reference interval (0.0–10.0 mg/dL) at both the 24 and 48 hour post-surgical samples, clearly documenting the presence of an active inflammatory process.

## Supporting information

S1 Appendix Surgical and anesthetic protocol. (DOCX)

S1 Fig Relationship between CRP levels pre- and post- medial patellar luxation surgery. (TIFF)

S2 Fig Relationship between WBC count pre- and post-medial patellar luxation surgery. (TIFF)

S1 Dataset Pre-anesthetic baseline hematology and chemistry results. (DOCX)

S2 Dataset Post surgical CRP and WBC counts. (PDF)
