# Supplementary material for: Characterization of C-reactive protein in dogs undergoing medial patellar luxation surgery
Source: PLoS One. 2020 May 8;15(5):e0231445. doi: 10.1371/journal.pone.0231445 (PMC7209118; doi:10.1371/journal.pone.0231445)
Supplement: S1 Appendix — (DOCX) [file pone.0231445.s001.docx]

The surgeries were performed by two individual surgeons. Premedication was standardized and consisted of 0.03 mg/kg acepromazine, 0.4 mg/kg methadone and 0.2 mg/kg midazolam, administered by intramuscular injection approximately 15-40 minutes prior to induction of anesthesia. Anesthesia was induced with propofol 1% 1-4 mg/kg, administered slowly intravenously before placement of an endotracheal tube, and the dogs were maintained in anesthesia with sevoflurane inhalation.

The hind limb was clipped and prepared aseptically for surgery. Local anesthetic was provided by 0.1 ml/kg bupivacaine administered as a femoral and sciatic nerve block guided by electrolocation. An incision in the skin was made over the medial side of the stifle, the surgical wound was redirected laterally and the parapatellar articular incision was made on the lateral side of the joint. The patella was then luxated medially to expose the femoral trochlea.

Trochleoplasty was performed as a trochlear block recession. An osteochondral autograft of the trochlear groove was harvested using a bone saw to create two abaxial osteotomies with an approximately 10-degree angle axially from each trochlear rim, and a basilar osteotomy was achieved using an appropriately sized osteotome and a mallet. The bloc was removed and cancellous bone was resected from the recipient bed or the block in order to achieve recession of the articular surface. The bloc was then carefully replaced and press-fitted against the recipient bed.

Subsequently a tibial tuberosity transposition was performed. A bone saw was positioned underneath the distal insertion of the patellar ligament and an incomplete osteotomy of the tibial crest was performed, leaving the distal periosteal attachment intact. The tibial crest was adjusted laterally and fixated using pin and tension band-wire. Finally, the joint capsule was imbricated laterally using an absorbable polyglyconate monofilament suture (Maxon®) in a horizontal mattress suture pattern. The fascia, subcutis and cutis were closed using a simple continuous suture pattern and polyglyconate/synthetic polyester monofilament absorbable (Maxon/Biosyn®) and nylon monofilament non-absorbable (Monosof®) suture respectively.

All dogs in the study were provided with a continuous rate infusion with
methadone (0.12 mg/kg/h), lidocaine (0.4 mg/kg/h) and ketamine (0.4 mg/kg/h), which was initiated at the beginning of surgery and continued until the next morning. The dogs were also given 0.2 mg/kg meloxicam. and 0.1 mg/kg maropitant post-operative. All dogs were administered meloxicam 0.1 mg/kg q 24 h and 5-10 mg/kg q 12 h gabapentin orally for a minimum of 10 days after surgery. None of the dogs received peri- or postoperative antibiotics.
