# Supplementary figures and images for: Characterization of C-reactive protein in dogs undergoing medial patellar luxation surgery
Source: PLoS One. 2020 May 8;15(5):e0231445. doi: 10.1371/journal.pone.0231445 (PMC7209118; doi:10.1371/journal.pone.0231445)

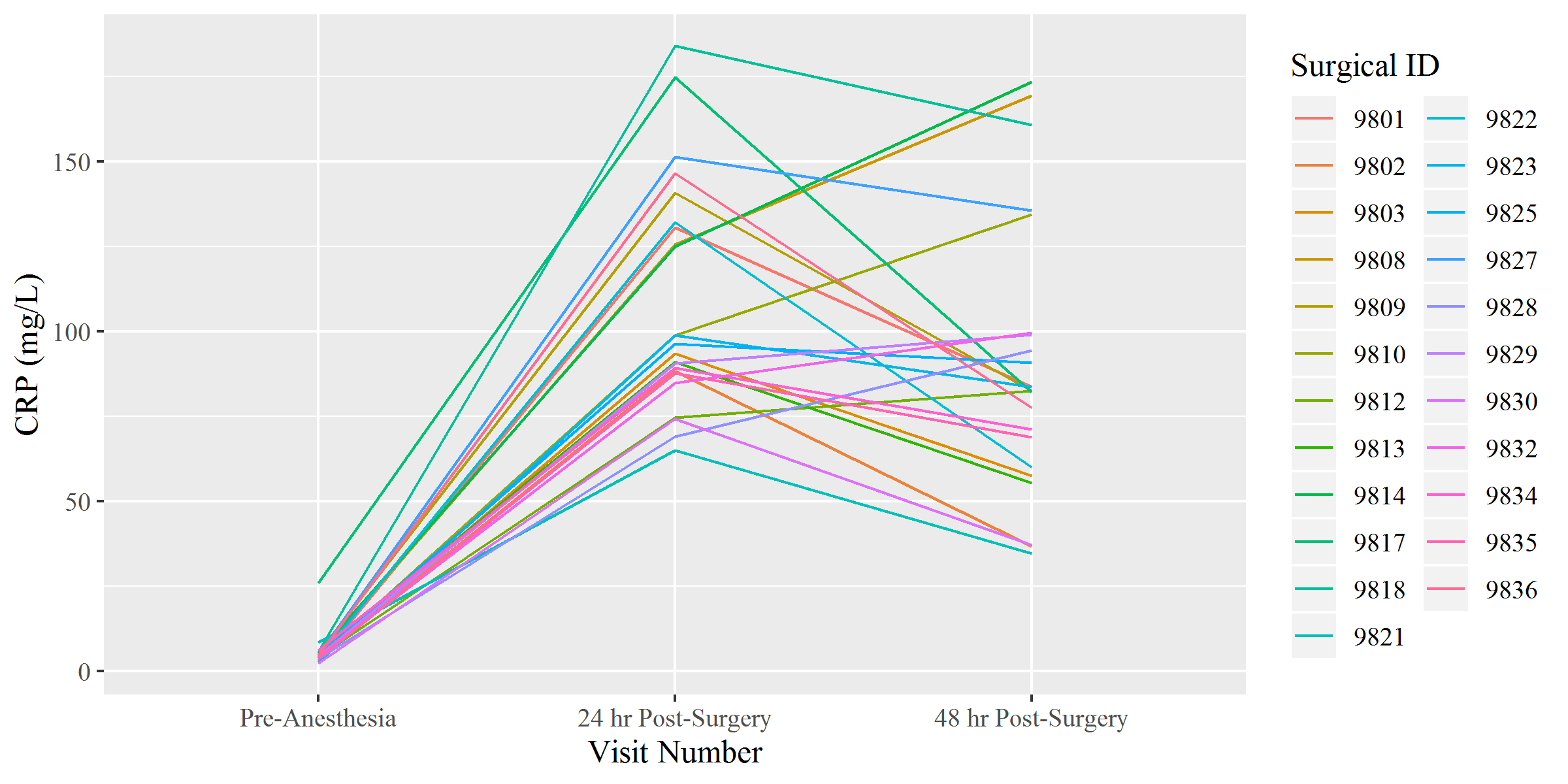

Supplement: S1 Fig — (TIFF) [file pone.0231445.s002.tiff]

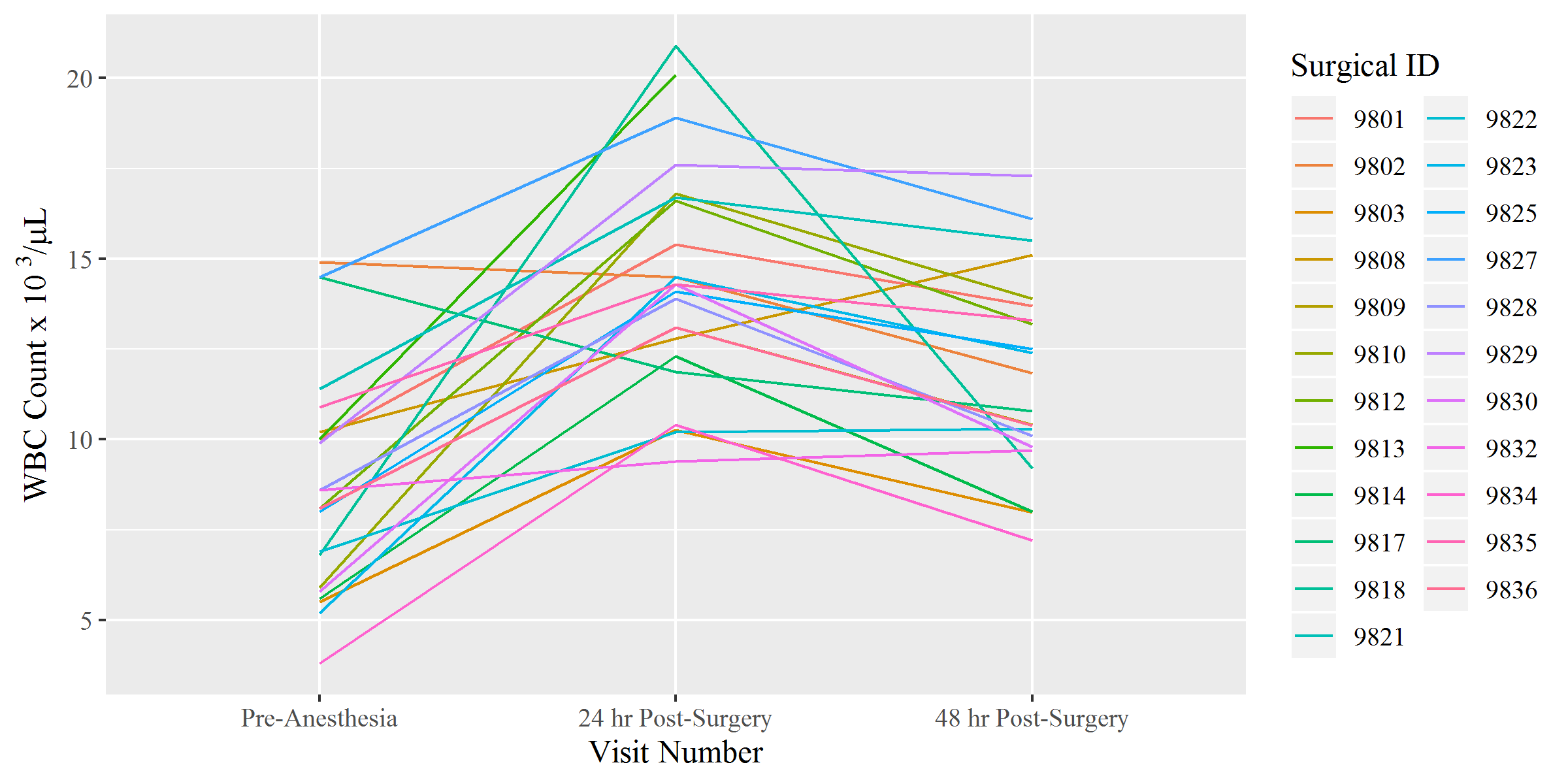

Supplement: S2 Fig — (TIFF) [file pone.0231445.s003.tiff]
