# Supplementary material for: Characterization of C-reactive protein in dogs undergoing medial patellar luxation surgery
Source: PLoS One. 2020 May 8;15(5):e0231445. doi: 10.1371/journal.pone.0231445 (PMC7209118; doi:10.1371/journal.pone.0231445)
Supplement: S1 Dataset — (DOCX) [file pone.0231445.s004.docx]

| Surgical ID | Sex | RBC *10^12^ /L | HCT L/L | HGB g/L | MCV fL | MCH pg | MCHC g/L | RDW % | Reticulocyte (absolute) K/uL | WBC *10^9^ /L | Neu *10^9^ /L | Lym *10^9^ /L | Mono *10^9^ /L | Eos *10^9^ /L | Baso *10^9^ /L | PLT *10^9^ /L | ALB g/L | BUN mmol/L | mmol/L | ALB/GLOB | BUN/CREA | TP g/L | ALP U/L | ALT u/L | CREA mol/L | GLOB g/L | CRP mg/L |
| --- | --- | --- | --- | --- | --- | --- | --- | --- | --- | --- | --- | --- | --- | --- | --- | --- | --- | --- | --- | --- | --- | --- | --- | --- | --- | --- | --- |
| **9801** | **MI** | **6.37** | **41.2** | **15** | **64.7** | **23.5** | **36.4** | **18.4** | **79** | **9.95** | **6.79** | **1.44** | **0.62** | **1.09** | **0.01** | **329** | **33** | **6** | **6.46** | **1** | **24** | **66** | **47** | **87** | **66** | **33** | **19.6** |
| **9802** | **MI** | **7.16** | **48.5** | **17.7** | **67.7** | **24.7** | **36.5** | **16** | **15.8** | **14.93** | **11.18** | **2.22** | **0.81** | **0.65** | **0.07** | **352** | **32** | **3.9** | **6.86** | **1** | **14** | **63** | **46** | **91** | **73** | **31** | **4** |
| **9803** | **FI** | **7.48** | **46.4** | **17.1** | **62** | **22.9** | **36.9** | **17.8** | **11.2** | **5.51** | **3.07** | **1.92** | **0.33** | **0.18** | **0.01** | **145** | **34** | **7.1** | **5.01** | **1** | **18** | **67** | **163** | **193** | **97** | **33** | **4.2** |
| **9808** | **FI** | **6.56** | **43.2** | **15.6** | **65.9** | **23.8** | **36.1** | **15.4** | **28.9** | **10.2** | **6.75** | **2.54** | **0.52** | **0.38** | **0.01** | **332** | **33** | **6.2** | **5.73** | **1.1** | **21** | **63** | **113** | **64** | **67** | **30** | **4.6** |
| **9809** | **FI** | **7.39** | **49.2** | **17.5** | **66.6** | **23.7** | **35.6** | **17.5** | **192.9** | **8.06** | **5.88** | **1.49** | **0.44** | **0.25** | **0** | **476** | **35** | **4.1** | **6.07** | **1.3** | **16** | **63** | **27** | **68** | **66** | **28** | **5.3** |
| **9810** | **MN** | **5.82** | **38.5** | **13.6** | **66.2** | **23.4** | **35.3** | **16.2** | **30.8** | **5.86** | **2.91** | **2.46** | **0.35** | **0.14** | **0** | **369** | **27** | **6.7** | **6.1** | **1** | **19** | **54** | **53** | **63** | **89** | **27** | **4** |
| **9811** |  |  |  |  |  |  |  |  |  |  |  |  |  |  |  |  |  |  |  |  |  |  |  |  |  |  |  |
| **9812** | **FI** | **5.81** | **39.2** | **14.4** | **67.5** | **24.8** | **36.7** | **14.8** | **37.8** | **8.11** | **3.9** | **3.31** | **0.54** | **0.36** | **0** | **229** | **32** | **6.2** | **6.36** | **1.3** | **15** | **57** | **27** | **23** | **96** | **25** | **4.2** |
| **9813** | **FI** | **6.16** | **39.3** | **14.5** | **63.8** | **23.5** | **36.9** | **14.6** | **45.6** | **9.99** | **4.88** | **3.75** | **1.19** | **0.17** | **0** | **678** | **31** | **5.8** | **5.21** | **1.1** | **27** | **60** | **38** | **42** | **49** | **29** | **5.5** |
| **9814** | **FI** | **6.93** | **45.8** | **16.1** | **66.1** | **23.2** | **35.2** | **16.7** | **17.3** | **5.55** | **3.09** | **1.61** | **0.32** | **0.51** | **0.02** | **241** | **33** | **9.4** | **4.43** | **1** | **22** | **67** | **54** | **57** | **110** | **34** | **5.3** |
| **9817** | **MI** | **7.66** | **48.1** | **17.1** | **62.8** | **22.3** | **35.6** | **20** | **83.5** | **14.47** | **10.33** | **2.5** | **1.39** | **0.25** | **0** | **339** | **34** | **12.7** | **7.07** | **1** | **72** | **68** | **174** | **115** | **46** | **34** | **26** |
| **9818** | **FI** | **8.31** | **58.5** | **20.6** | **70.4** | **24.8** | **35.2** | **18** | **172** | **6.77** | **4.33** | **1.51** | **0.41** | **0.49** | **0.03** | **325** | **34** | **4.2** | **2.21** | **1** | **15** | **68** | **51** | **42** | **72** | **34** | **5.7** |
| **9821** | **FI** | **7.29** | **46.4** | **16.5** | **63.6** | **22.6** | **35.6** | **16.1** | **36.5** | **11.44** | **7.15** | **3.1** | **0.6** | **0.58** | **0.01** | **457** | **32** | **5.1** | **5.75** | **0.8** | **18** | **72** | **46** | **57** | **73** | **40** | **8.5** |
| **9822** | **MI** | **7.06** | **44.4** | **15.9** | **62.9** | **22.5** | **35.8** | **18.8** | **81.2** | **6.88** | **4.3** | **1.66** | **0.6** | **0.31** | **0.01** | **348** | **29** | **10.3** | **5.4** | **1** | **36** | **58** | **204** | **157** | **73** | **29** | **3.7** |
| **9823** | **FI** | **7.57** | **48** | **17.2** | **63.4** | **22.7** | **35.8** | **19.4** | **-** | **5.24** | **3.32** | **1.4** | **0.3** | **0.22** | **0** | **341** | **33** | **3.5** | **5.13** | **0.8** | **13** | **73** | **156** | **140** | **69** | **40** | **2.6** |
| **9825** | **MI** | **5.6** | **34.1** | **12.6** | **60.9** | **22.5** | **37** | **16.2** | **37.5** | **7.96** | **4.45** | **2.22** | **0.91** | **0.38** | **0** | **279** | **28** | **5.3** | **7.3** | **1** | **25** | **56** | **90** | **73** | **50** | **28** | **4.4** |
| **9827** | **FI** | **6.75** | **43.3** | **15.7** | **64.1** | **23.3** | **36.3** | **14.5** | **20.3** | **14.47** | **7.93** | **4.62** | **1.1** | **0.81** | **0.01** | **392** | **32** | **4.5** | **6.71** | **1.1** | **19** | **62** | **61** | **45** | **61** | **30** | **4.7** |
| **9828** | **FI** | **6.36** | **42.8** | **15.7** | **67.3** | **24.7** | **36.7** | **16.6** | **54.7** | **8.62** | **5.99** | **1.61** | **0.63** | **0.38** | **0.01** | **372** | **33** | **4.1** | **5.3** | **0.9** | **12** | **70** | **56** | **58** | **81** | **37** | **3.7** |
| **9829** | **FI** | **8.64** | **54.6** | **18.7** | **63.2** | **21.6** | **34.2** | **19.7** | **82.1** | **9.87** | **6.83** | **1.99** | **0.43** | **0.6** | **0.02** | **414** | **38** | **6.2** | **6.14** | **1.3** | **13** | **68** | **50** | **39** | **115** | **30** | **4.4** |
| **9830** | **MN** | **6.34** | **44.3** | **16.3** | **69.9** | **25.7** | **36.8** | **14.6** | **16.5** | **5.77** | **3.95** | **1.12** | **0.3** | **0.39** | **0.01** | **186** | **33** | **5.3** | **6.33** | **1** | **14** | **65** | **24** | **61** | **100** | **32** | **2.2** |
| **9832** | **MI** | **7.85** | **47.7** | **17.8** | **60.8** | **22.7** | **37.3** | **20.9** | **71.4** | **8.59** | **5.09** | **2.18** | **0.81** | **0.38** | **0.13** | **362** | **29** | **5** | **6.49** | **1** | **18** | **59** | **42** | **65** | **69** | **30** | **4.2** |
| **9834** | **FI** | **7.56** | **47.5** | **17.3** | **62.8** | **22.9** | **36.4** | **17** | **47.6** | **3.78** | **1.6** | **1.65** | **0.33** | **0.19** | **0.01** | **330** | **30** | **7.1** | **8.51** | **0.9** | **29** | **62** | **85** | **72** | **62** | **32** | **6.1** |
| **9835** | **FI** | **7.02** | **45.2** | **16.3** | **64.4** | **23.2** | **36.1** | **18.6** | **62.5** | **10.88** | **5.81** | **4.03** | **0.75** | **0.29** | **0** | **344** | **31** | **8.2** | **5.7** | **1.1** | **26** | **60** | **43** | **124** | **79** | **29** | **3.7** |
| **9836** | **FI** | **6.58** | **43.7** | **16.5** | **66.4** | **25.1** | **37.8** | **16.4** | **122.4** | **8.01** | **5.54** | **1.48** | **0.75** | **0.24** | **0** | **608** | **39** | **8.2** | **5.95** | **1** | **33** | **79** | **11** | **105** | **62** | **40** | **4.6** |
