# Supplementary material for: Characterization of C-reactive protein in dogs undergoing medial patellar luxation surgery
Source: PLoS One. 2020 May 8;15(5):e0231445. doi: 10.1371/journal.pone.0231445 (PMC7209118; doi:10.1371/journal.pone.0231445)
Supplement: S2 Dataset — (PDF) [file pone.0231445.s005.pdf]

#	usubjid	crp0	crp1	crp2	Date.start	wbc0	wbc1	wbc2	minutes.surgery	weight	bcs
1	9801	3.1	130.5	83.6	09.04.2017	10	15.4	13.7	40	6.77	5
2	9802	4	88.2	36.7	04.05.2018	14.9	14.49	11.84	35	3.86	5
3	9803	4.2	93.5	57.5	16.04.2018	5.5	10.27	7.98	38	5.6	5
4	9808	4.6	125.6	169.4	21.3.2017	10.2	12.8	15.1	24	3.31	4
5	9809	5.3	140.7	82.5	22.06.2017	8.1	13.1	10.4	NA	5.9	5
6	9810	4	98.8	134.4	07.02.2018	5.9	16.8	13.9	36	2.9	6
7	9812	4.2	74.6	82.4	18.05.2017	8.1	16.6	13.2	NA	15.1	7
8	9813	5.5	90.9	55.4	07.12.2018	10	20.1	NA	32	1.62	5
9	9814	5.3	124.9	173.5	11.04.2018	5.6	12.3	8	45	30.6	5
10	9817	26	174.7	82	19.09.2017	14.5	11.87	10.79	35	2.3	5
11	9818	5.7	184.1	160.8	20.09.2017	6.8	20.9	9.2	38	3.3	6
12	9821	8.5	65	34.5	22.09.2017	11.4	16.7	15.5	NA	4.9	6
13	9822	3.7	132.1	59.9	07.11.2017	6.9	10.2	10.3	40	6.1	6
14	9823	2.6	98.8	83.6	14.09.2017	5.2	14.5	12.4	29	5.6	6
15	9825	4.4	96.4	90.8	30.08.2018	8	14.1	12.5	40	3	5
16	9827	4.7	151.4	135.6	11.10.2017	14.5	18.9	16.1	NA	NA	NA
17	9828	3.7	69.1	94.4	15.02.2018	8.6	13.9	10.1	NA	8.5	6
18	9829	4.4	90.5	99	21.04.2017	9.9	17.6	17.3	37	6.5	5
19	9830	2.2	74.3	37	11.04.2018	5.8	14.3	9.8	NA	19	6
20	9832	4.2	84.8	99.6	09.01.2018	8.6	9.4	9.7	NA	9.5	6
21	9834	6.1	89.3	71.2	27.03.2018	3.8	10.4	7.2	NA	2.2	5
22	9835	3.7	87.6	68.8	13.06.2017	10.9	14.3	13.3	32	4.5	5
23	9836	4.6	146.6	77.5	11.04.2018	8.1	13.1	10.39	NA	5.5	5
